# The Anti-Tuberculosis Crusade and Phthisiophobia*Read before the Graduate Nurses’ Association, November 3, 1908.

**Published:** 1909-05

**Authors:** Theo. Y. Hull

**Affiliations:** San Antonio, Texas


THE
### TEXAS MEDICAL JOURNAL.
Established July, 1885.

F. E. DANIEL, M. D., - - - - Editor, Publisher and Proprietor

PUBLISHED MONTHLY.—SUBSCRIPTION $1.00 A YEAR.

VOL. XXIV. AUSTIN, MAY, 1909. No. 11.

The publisher is not responsible for the views of contributors.

### Original Articles.

For Texas Medical Journal.

### The Anti-Tuberculosis Crusade and Phthisiophobia.*

BY THEO. Y. HULL, M. D., SAN ANTONIO, TEXAS.

The twenty-eighth day of September should be set apart in this country as a day to commemorate what may prove one of the greatest events in our history, the opening of the sixth triennial meeting of the International Congress on Tuberculosis at Washington.

This day witnessed the inauguration of one of the greatest educational campaigns on record in the United States or elsewhere, and from which we expect the most far-reaching results. It marks the opening in this country of "humanity's crusade against the white plague." Forty-five hundred delegates were assembled in "humanity's name" and for "humanity's sake." Practically every civilized country in the world was represented officially.

Secretary Cortelyou, in his opening address in behalf of the United States government, spoke of the importance of the movement. The convention was characterized as a Peace Congress of more importance than that of The Hague, inasmuch as its efforts were directed against an ever-present and persistent foe, and that all nations, without thought of boundaries, were united in seeking the common good; a war congress, inasmuch as the greatest modern war is against tuberculosis, and will test our resourcefulness and our modern strategy.

*Read before the Graduate Nurses' Association, November 3, 1908.

It was predicted that when the doors were closed, October 12th, it would be voted one of the greatest accomplishments of the twentieth century, "the era of big things," and it was. Thus began and thus ended the International Congress on Tuberculosis, the battle of words ended and the battle of deeds began. It is now the time to "do things." In the drama enacted—I might say tragedy—and yet to be enacted, you and I each have our part. The movement is forward. There will be no looking back. The tragedy of Lot's wife will not be re-enacted.

The anti-tuberculosis fight has two phases, both of which we may and must participate in. The one deals with the sick, which, like the poor, are ever with us, and which we must treat and care for as a professional duty; and the other takes up the prevention of tuberculosis as a social obligation.

In both phases of the problem, the nurse and the physician may find important work to do. We should no more think of shrinking from our duty than the soldier from going into battle, where death may be the reward of his valor. Many of us may, and some of us doubtless will, receive the same reward for our devotion to a cause, but there are some things worse than *death*.

The part of the nurses and their organizations in this crusade is still in its beginning, but it was thought of sufficient importance in the Congress to set aside one day in one of the sections for the discussion of their work. As far back as 1877, the Visiting Nurses' Association was organized. In many cities nurses were trained under the auspices of the church to do visiting nurses' work among the poor. In 1893 two nurses of the Henry Street Settlement, New York, were sent out on visits of instruction to the homes of patients. A part of their work was to leave sputum cups and disinfectants, and to teach their use. In 1900, the "follow-up work" was organized in Baltimore in co-operation with the Charity Organization Society. In 1902-3 the Tuberculosis Committee of the New York Charity Organization Society employed special nurses for this work. And so the work has gone on in one city after another. Hospital training schools for nurses are teaching and drilling these "soldiers in the field" and they are true soldiers doing the bravest of brave things—facing an invisible foe.

The Henry Phipps Institute in Philadelphia is doing a great work, and here nurses are especially trained for the tuberculosis department. They are employed in the "follow-up work" to see that patients carry out the rules of the institute, to explain these rules where they are not understood, and to encourage the sick in doing better.

I do not blame any nurse for not seeking this field of work. It is not pleasant, and very often is not profitable, but some one must do it. Any of you are liable to be called upon to do so any day, especially in this city, where there are probably more than two thousand active cases. Only the physically strong should undertake it, and those who have once been infected should be exceedingly careful about reinfection. Fortunately for the nurse, most of those who employ her are able to furnish the things necessary to make everything sanitary and cleanly, and intelligent enough to follow all reasonable instructions and take all necessary precautions. When this is done, there is practically little more danger in handling such a patient than any other. When the patient will not do the things necessary to protect others, there is but one thing to do—leave. Your own life is worth something, and should be so regarded. No amount of money can repay you for lost health.

### WHAT DOES TUBERCULOSIS MEAN IN THE UNITED STATES?

It simply means, in plain language, that there are today between one and two million active cases, making that many centers for the further propagation of the disease. Fully 75 per cent of this number must die, as they have already passed beyond the point where human aid is available. It means that two hundred thousand persons die annually from this one cause within the continental confines of this country, fully 75 per cent of whom are wage-earners, that is, persons who, in a condition of health, would be the makers and supporters of homes. It means from two to four years of comparative invalidism, and from one to two years of complete invalidism, with all the concomitant evils—of sorrow, of suffering, and of misery, and hopeless existence for each of these two hundred thousand victims. It means that there is charged up annually against the prosperity of this country for the loss of human life, for the loss of wage-earnings, and for various other items incident to the period of invalidism and death, a bill for about $1,500,000,000, a sum nearly equal to our national debt, and there is still other and greater losses upon which no estimate can be placed in dollars and cents. It means, in a senes, racial degeneration, for in this life the victory is to the strong, and the children of tubercular parents are handicapped by birth. The difference between success and failure is only too often the difference between inherited predisposition and tendencies. It means greater or less social destruction, with the blighting of hopes, the breaking up of homes, and the sundering of all the ties that are dear to us. It means an increase in crime, for who knows how many write their names in our criminal records? It means that every one of us is exposed to the contagion continually, and while we seek to escape it in one place, we are apt to encounter it in another. We are reaping the harvest prepared by our fathers, mostly in ignorance of the simple laws of health. Will we, in the light of our knowledge, leave a better inheritance to our children? We hope so. The future generations have an inherent right to the full benefits of the accumulated knowledge of the past generations.

### HOW IS TUBERCULOSIS CONTRACTED?

A knowledge of the source of infection is important, as all our means of prevention rest upon it. The specific agent of infection is the tubercle bacillus. Its source is the excreta—sputum, feces, urine, etc., of the tubercular person or animal, cattle, etc. The growth of the tubercle bacillus within the tissues of the body is the cause of tuberculosis. There can be no tuberculosis without the bacillus. The disease is not inherited, it is contracted. The chief channels of infection are the air we breathe and the food we eat. It may be of interest to note the change in our ideas of the channels of infection. A few years ago, tuberculosis was thought to be largely inherited. Now we know that cases of supposed inheritance were due to direct infection of the child by the tuberculous parent. With inheritance disproven, another step was gained. Then the knowledge that the germs could be carried directly into the lungs with the air we breathe led to the belief that this was the chief channel of infection. Many experiments and the most frequent location in the apices of the lungs tended to confirm this belief. Late experiments carried out in Sweden tend strongly in the same direction. The discovery of a practical identity of human and bovine tuberculosis, together with the frequency of tubercular infection in cattle, has centered the attention of the scientific world upon the milk and meat supply, and the possibilty of the gastro-intestinal tract being the chief channel of infection. This idea was further strengthened by the discovery that tubercle bacilli taken up by the blood current would be deposited most frequently in the apices of the lungs. In the light of this fuller knowledge, we are in a position to study intelligently the next question.

### HOW MAY WE PREVENT TUBERCULOSIS?

With the certainty that tuberculosis is contracted and not inherited comes the further certainty that it may be prevented, and what may be prevented will be in time. There was a time when smallpox carried off one-tenth of the people, but today it is practically unknown in many places. In less than fifty years we expect to put tuberculosis in the list of diseases conquered. The watchword in the fight will be *"prevention."* We must bear in mind another fact, that tuberculosis, while infectious, is also contagious; that is, that it is possible to take the disease by direct contact. This throws light upon the extreme frequency of the disease among the children of tuberculous parents, and also that this frequency increases with the age and opportunity of infection. Dr. Sachs has given us some interesting data on this point on his studies on the frequency of tuberculosis among the children of laboring classes of Chicago. In 1800 autopsies held on children, dead from other causes, over 20 per cent had tubercular infection, the per cent increasing with the age of the children (under one year, 7.1 per cent; one to two years, 16 per cent; two to four years, 24.5 per cent; four to six years, 26.9 per cent; six to eight years, 26.8 per cent). Among the living children of tubercular parents, 53.1 per cent were proven to be infected. In these cases direct contact must have been a powerful factor. Tubercle bacilli have been found on the hands and under the finger nails of children. With the child's most frequent position upon the floor, and his known tendency to put everything his hand touches into his mouth, much may be explained. Here is where ignorance becomes criminal, and carelessness is equivalent to murder. It is possible to cite case after case in my own experience where ignorance and carelessness have wrought destruction. This is one place where cleanliness is greater than godliness.

Let me impress on you the idea of absolute cleanliness. It may save your life and that of others. We must remember that the contagious element—the bacillus—is contained in the excreta, chiefly the sputum; but we should not forget that it may be found in the feces and the urine before it appears in the suptum. If the sputum, etc., are completely destroyed, there can be no danger to others. The care of the sputum is important. No patient should be permitted to expectorate into an open receptacle. The danger from flies, etc., is too great. I prefer that every patient should use a metallic sputum cup, with lid, and specially prepared paper cups, so that the sputum may be destroyed as often as necessary, and the metallic cup boiled. Every patient should be provided with small paper napkins and paper bags when out of doors, or in places where he does not wish to carry a cup; the napkins may be used to receive the expectoration. It should be immediately folded and placed in the paper bag, to be burned later. The lips should be wiped with the napkin after expectoration, and the hands frequently washed. Again, the patient should be taught when coughing or sneezing to hold something in front of his mouth to catch stray particles of sputum. This is essential to perfect cleanliness, but is often overlooked.

Now, a few words as to the room. It should be light, well ventilated, cheerful, and without carpet. Only small rugs may be used, such as may be placed in the direct sunlight frequently. Everyone seems to know why we want the room well ventilated, although many fail to carry it out. But in regard to light, there seems to be some doubt—a sort of photophobia. Here is a fact we should not soon forget. The sunlight is a powerful disinfecting agent, and the direct rays will kill the tubercle bacillus in a few seconds. Dust has been taken from a properly lighted room occupied by a tubercular patient and found free from bacilli, while dust taken from the walls of churches and theaters has been found literally alive with virulent bacilli. The bedding should be chiefly such as can be boiled, and frequently placed in the sunlight. The room should be thoroughly disinfected when vacated, and the wood-work should be frequently washed with antiseptic solution while occupied. It may not be possible to render rooms aseptic, but they can be rendered free of danger to others. Tubercular patients need not be a menace to any one, and if a few simple rules are observed need not endanger the life of nurse, family or friends. It is only through ignorance and willful neglect that the sick become dangerous to others.

One of the most powerful agencies for the prevention of tuberculosis is compulsory registration. In cities where it has been carried out for some time it has proven a powerful agent in awakening the public mind, and of giving aid and instruction where it is needed. It is not intended to annoy and harass the sick, nor to make their burden more unbearable. It is not intended to hold the unfortunate victim up before the public, and does not. It is simply intended to give the Board of Health, and the Board of Health only, the knowledge of the existence of the disease, that in case the attending physician does not assume the responsibility of instructing his patient in preventive measures, it may do so. The results in other cities have been most beneficial. In these Southwestern cities to which so many tubercular people flock, and in which the mortality from tuberculosis is so appalling, some active measures are imperative. In this work of popularizing registration, the nurses and their association can render valuable assistance.

There is another field in which you can work with great effect. You have access to the Mothers' Clubs and other societies for the betterment of womankind, and, therefore, of all mankind. You can preach there the gospel of a healthier life, and, therefore, a better life. You can teach the doctrines of fresh air, of pure food, and of cheerful homes. In the light of sanitary surroundings and wholesome living tuberculosis will lose many of its terrors.

There is another field in which you can help. We intend to carry the work into the public and private schools, and organize the children into an army of effective workers. We do not expect to save the present generation—their fate is sealed. We do expect, by properly educating the youth of the country in matters of this kind, to greatly advance the work and eventually conquer the disease. I am aware that there are those who are not in sympathy with this movement, and, unfortunately, many of them are found in the ranks of the medical profession. If the crusade were against any other form of evil they would probably raise the same objections. The fact is that the world is moving on, and into newer and better things. The crusade against tuberculosis will continue until its object is accomplished, and may we each and every one of us add our mite toward the speedy coming of that day.

*Phthisiophobia.*—There is an old saying that familiarity breeds contempt, and it is a common observation that those accustomed to danger think little of it. For hundreds of years tuberculosis has claimed its victims by the thousands in every country. No community has been exempt. The most common experience has been the slow wasting away of some relative or friend or neighbor, and so it has come to pass that the people had accepted the ravages of tuberculosis as a matter of course. But all earthly things have an end, and sometimes it happens that the end iis ushered in by a sudden revulsion of feeling. It has so happened in tuberculosis. A full realization of its menace has suddenly burst upon the people, and, in some communities a blind, unreasoning fear has taken the place of apathy. It is this fear that I have termed phthisiophobia, a most unwelcome apparition known as the "consumption terror" In some places it has almost paralyzed the struggle against tuberculosis, by rendering it almost impossible for tubercular people to care for themselves. If the victim is truthful, he finds it difficult to obtain a dwelling place, and still more difficult to obtain employment because of the fear of the malady among his fellow workmen. The fear of disclosure often prevents him from receiving advice and treatment at the moment it would be of the greatest value to him, and also operates against his taking necessary measures to prevent others from contracting the disease.

The indignities suffered by some through the heartlessness, cowardice and ignorance of their fellow creatures is worse than the disease itself. The invalid often finds himself a veritable leper in a Christian land, and I doubt that the lepers of old without the city walls felt the stigma more keenly.

The registration of cases of tuberculosis is opposed largely for this reason, and it is not uncommon to hear physicians advancing the same objections. In this manner laws and ordinances, the enforcement of which would result in great good to the community, are brought to naught.

A little knowledge of facts in regard to tuberculosis, and a little reasoning therefrom might dispel much of this terror. In the present state of society, no one can escape coming in contact with the tubercle bacilli. Infection takes place, however, only under conditions of lowered vitality. The main source of danger is the tubercular sputum, and this should always be destroyed. Tuberculosis can not be contracted like the acute infectious diseases, by merely coming in contact with the infected person. Neither is the person who does not cough nor expectorate dangerous, and the use of proper precautions by those who do, minimizes the danger therefrom. The natural defensive powers of the healthy adult organism largely diminishes the chances of infection. Moreover, the belief that tuberculosis is chiefly contracted during childhood is gaining ground.

Finally, where there is absolute cleanliness, there is no contagion; and may we, as physicians and nurses, work unceasingly to attain that ideal condition.

